# Exposed implants five years after spine surgery: poor outcomes due to poor follow-up

**DOI:** 10.11604/pamj.2021.40.173.30046

**Published:** 2021-11-21

**Authors:** Tapan Patel, Shivani Patel

**Affiliations:** 1 Department of General Surgery, Baroda Medical College, Vadodara, India

**Keywords:** Spinal tuberculosis, exposed implants, implant extraction, patient follow-up, patient care

## Image in medicine

An eighteen-year-old female presented with the complaint of dull aching lower back pain for past 3 months. The pain was localized and was mildly aggravated by movement. She had a history of resolved spinal tuberculosis and vertebral fracture managed with pedicle screw fixation and L1-L3 vertebral fusion before five years. She had not followed up since then. On examination, there were exposed and protruding metallic implants surrounded by dried and foul smelling tissue in the lumbar area of the back (A). The skin surrounding it was normal. Lateral X-ray suggested that the screws were retracted from their original site (B). There were no signs of infection of the deeper tissues. Her vitals were normal. Hematological parameters were suggestive of mild iron deficiency anemia. Her condition was managed by surgical extraction of the implants and debridement of the damaged tissue. She was discharged after one week and her follow-up after two weeks was normal. The most important factor to be considered here is the lack of follow-up appointments. The probable reasons behind the lack of follow-up in our patient are suspected to be lack of patient education and poor socio-economic status. These factors play a major role in patient care, especially in the developing countries.

**Figure 1:**
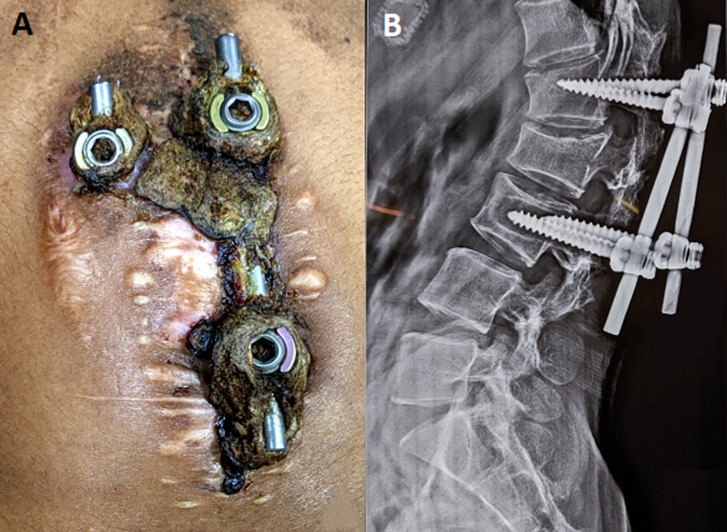
A) exposed implants protruding from lumbar region, surrounded by dry necrotic tissue; B) X-ray lateral view of the lumbar spine suggestive of retracted implants

